# Towards a model of postglacial biogeography in shallow marine species along the Patagonian Province: lessons from the limpet *Nacella magellanica* (Gmelin, 1791)

**DOI:** 10.1186/1471-2148-12-139

**Published:** 2012-08-07

**Authors:** Claudio A González-Wevar, Mathias Hüne, Juan I Cañete, Andrés Mansilla, Tomoyuki Nakano, Elie Poulin

**Affiliations:** 1 Laboratorio de Ecología Molecular, Instituto de Ecología y Biodiversidad (IEB), Departamento de Ciencias Ecológicas, Facultad de Ciencias, Universidad de Chile, Las Palmeras # 3425, Ñuñoa, Santiago, Chile; 2 Departamento de Recursos Naturales, Universidad de Magallanes, Punta Arenas, Chile; 3 Seto Marine Biological Laboratory, Field Science Education and Research Centre, Kyoto Univeristy, 459 Shirahama, Nishimuro, Wakayama, 649-2211, Japan

**Keywords:** Quaternary, Cape horn current, Last glacial maximum, Post-glacial recolonization, Expansion-contraction model, *Nacella magellanica*, Larval dispersal, Asymmetric gene flow, Patagonian Province, Falkland/Malvinas Islands

## Abstract

**Background:**

Patagonia extends for more than 84,000 km of irregular coasts is an area especially apt to evaluate how historic and contemporary processes influence the distribution and connectivity of shallow marine benthic organisms. The true limpet *Nacella magellanica* has a wide distribution in this province and represents a suitable model to infer the Quaternary glacial legacy on marine benthic organisms. This species inhabits ice-free rocky ecosystems, has a narrow bathymetric range and consequently should have been severely affected by recurrent glacial cycles during the Quaternary. We performed phylogeographic and demographic analyses of *N. magellanica* from 14 localities along its distribution in Pacific Patagonia, Atlantic Patagonia, and the Falkland/Malvinas Islands.

**Results:**

Mitochondrial (COI) DNA analyses of 357 individuals of *N. magellanica* revealed an absence of genetic differentiation in the species with a single genetic unit along Pacific Patagonia. However, we detected significant genetic differences among three main groups named Pacific Patagonia, Atlantic Patagonia and Falkland/Malvinas Islands. Migration rate estimations indicated asymmetrical gene flow, primarily from Pacific Patagonia to Atlantic Patagonia (N_e_m=2.21) and the Falkland/Malvinas Islands (N_e_m=16.6). Demographic reconstruction in Pacific Patagonia suggests a recent recolonization process (< 10 ka) supported by neutrality tests, mismatch distribution and the median-joining haplotype genealogy.

**Conclusions:**

Absence of genetic structure, a single dominant haplotype, lack of correlation between geographic and genetic distance, high estimated migration rates and the signal of recent demographic growth represent a large body of evidence supporting the hypothesis of rapid postglacial expansion in this species in Pacific Patagonia. This expansion could have been sustained by larval dispersal following the main current system in this area. Lower levels of genetic diversity in inland sea areas suggest that fjords and channels represent the areas most recently colonized by the species. Hence recolonization seems to follow a west to east direction to areas that were progressively deglaciated. Significant genetic differences among Pacific, Atlantic and Falkland/Malvinas Islands populations may be also explained through disparities in their respective glaciological and geological histories. The Falkland/Malvinas Islands, more than representing a glacial refugium for the species, seems to constitute a sink area considering the strong asymmetric gene flow detected from Pacific to Atlantic sectors. These results suggest that historical and contemporary processes represent the main factors shaping the modern biogeography of most shallow marine benthic invertebrates inhabiting the Patagonian Province.

## Background

Climatic changes are considered as one of the main factors regulating abundance, composition, and distribution of species at different temporal and spatial scales [1-3]. Direct historical evidence from fossil records indicates that many terrestrial species underwent rapid latitudinal shifts during the Quaternary glacial period and especially after the Last Glacial Maximum (LGM) around 23 to 18 ka [4-6]. Paleontological and palynological records from the Northern Hemisphere, along with biogeographic evidence, provided the empirical basis for the Expansion-Contraction (EC) model of Pleistocene biogeography [7] which describes the response of populations and species to climatic changes [3,4,6,8,9]. Under a basic EC model, cool-temperate species from the Northern Hemisphere survived the LGM at lower latitude refugia and then recolonized higher latitudes through range expansion [6,7].

The application of molecular-based approaches in population genetics has provided new insights into the history of many species and helped us to further understand the impact of the Quaternary glacial cycles on patterns of genetic variation and structure [2,3,6,7]. However, most examples come from studies of Northern Hemisphere biota mainly of terrestrial species [10,11]. In marine ecosystems, interglacial period deposits are rich in fossils but glacial records are in most cases unavailable due to the Holocene rise in sea level [3]. Phylogeographical studies in non-tropical areas of the Southern Hemisphere are scarce [12], but during the last decade more data has been accumulated in different southern South American groups [13-20]. Periodic global cooling during Quaternary glacial cycles (1.8 Ma – 10 ka) generated shifts in climate, landscape and sea level. For instance, during the LGM an ice sheet about 1800 km long covered the west slope of the Andes from 35°S to almost 56°S [21-24]. Much of the Atlantic side of Patagonia and northeastern Tierra del Fuego remained unglaciated through the late Pleistocene [21]. These glacial changes in Patagonia led to regional isolations and local extinctions, shaping the current patterns of species diversity in temperate areas of southern South America [13,16,25,26]. Genetic evidence of postglacial recolonization has been found in several Patagonian groups including galaxiid [16,26,27] and percichthyd fishes [13,14,28], lizards [29-31], amphibians [32], mammals [11,20,33-35] and plants [15,18,36]. These studies have provided conflicting results, indicating either postglacial colonization from restricted glacial refugia [26,32,36], recolonization from geographically distant ice-free regions [15,34], or local persistence through glacial cycles [16,28,31,33,36]. Few genetic studies have examined the effect of the Quaternary glacial cycles in marine organisms of southern South America and most of these were restricted to Pacific sectors of Patagonia [15,17,37-39]. Moreover, due to logistic problems especially in the hard-to-access fiordal region of Chilean Patagonia, most of these studies present unbalanced sampling, only including localities from easy-access areas which represent the northern (Reloncaví Archipelago and Chiloé Island) and the southern extremes of Patagonian species distributions (Magellan Strait and Tierra del Fuego).

The true limpet genus *Nacella* (Patellogastropoda: Nacellidae) includes 15 nominal species distributed in different biogeographical provinces of the Southern Ocean [40]. Along the Patagonian coast, *Nacella* represent a dominant group of benthic macro-invertebrates, especially in the marine rocky ecosystems [41-44]. Based on morphological characters, at least eight nominal species of the genus have been described in this region (*Nacella chiloensis*, *N. deaurata*, *N. delicatissima*, *N. flammea*, *N. fuegiensis*, *N. magellanica*, *N. mytilina*, and *N. venosa*; [40,43]. On the basis of species richness, Powell [40] considered Patagonia as the center of origin and diversification of *Nacella*, from where it expanded eastward through the West Wind Drift (WWD). However, this assumption has been recently rejected by phylogenetic reconstructions showing that the Patagonian group of *Nacella* is the most derived one and diversified no more than 2.0 Ma [45]. Molecular and morphological comparisons of Patagonian species suggest that the number of nominal species in *Nacella* was overestimated [17,46]. For instance, González-Wevar *et al.*[17] using COI sequences and geometric morphometrics in seven sympatric nominal species recognized only four units of *Nacella* in Patagonia. In spite of the absence of reciprocal monophyly [45], morphological, genetic and habitat preference differentiation among congeners are maintained even in sympatry. Considering these results, the diversification of *Nacella* in Patagonia includes four Evolutionarily Significant Units (ESUs): *N. deaurata*, *N. flammea*, *N. mytilina* and *N. magellanica*[17].

*Nacella magellanica* (Gmelin, 1791) exhibits the widest distribution in Patagonia, extending from Puerto Montt in the Pacific (42°S) to the Buenos Aires Province in the Atlantic (35°–40°S), including the Strait of Magellan, Cape Horn, Tierra del Fuego and the Falkland/Malvinas Islands [40,43]. This species is the most abundant and conspicuous limpet in intertidal and shallow subtidal areas of Patagonia [47]. It has been also reported in the Beagle Channel as an organism associated with holdfasts of the macroalga *Macrocystis pyrifera*[48]. As in other nacellid limpets, *N. magellanica* is a broadcast spawner that reproduces during austral spring [47] but its free-living larval duration is still unknown. A recent phylogeographic study of the species in Atlantic Patagonia identified an absence of genetic structure and a very recent geographic-demographic expansion (~ 11 ka) [49]. Nevertheless, there is still an absence of knowledge about the patterns of genetic diversity, structure and connectivity of the species in Pacific Patagonia.

The southern tip of South America constitutes an interesting system to evaluate the relative effects of habitat discontinuities, oceanography, and glaciological history in marine benthic organisms with limited autonomous motility that exhibit some degree of larval dispersal. The presence of an extensive ice sheet during the LGM in this region likely eradicated many populations of shallow-water marine benthic organisms. Considering the current distribution of *N. magellanica*, its narrow bathymetric range and its high abundance along both sides of Patagonia, this species constitutes a suitable model to infer how historical and contemporary climatic events shaped the patterns of population genetic diversity and structure. We analyzed samples from a total of 14 localities encompassing most of the species range in Pacific Patagonia and Tierra del Fuego, as well as two population from Atlantic Patagonia and individuals from the Falkland/Malvinas Islands. We aimed to test the hypothesis that (i) *N. magellanica* in the Pacific sector of Patagonia represents a post-glacial recolonization from restricted glacial refugia in the northern limit of its distribution, or alternatively, (ii) *N. magellanica* persisted unaffected through glacial cycles in this area. Also, we aimed to determine if glacial-interglacial periods promoted genetic differentiation or even divergence between Atlantic and Pacific populations. Finally, considering the glaciological history of Patagonia and the pattern of genetic diversity and structure in the species it will be possible to evaluate the role of the Falkland/Malvinas Islands in the phylogeography of the species as a source or sink area.

## Results

We analyzed a total of 357 individuals; the COI sequence data set consisted of 671 nucleotide positions coding 223 aminoacids. As expected for coding regions, no indels or stop codons were detected, sequences were not saturated at any position and no amino acid substitution was detected using the invertebrate mitochondrial table [50]. In the whole data set, *Nacella magellanica* exhibited intermediate levels of genetic diversity with 58 polymorphic characters (8.6%); 37 of them (5.5%) were parsimoniously informative. As previously estimated for nacellids [17,51], *N. magellanica* sequences were A-T rich (61.6%) compared to mean G-C content (39.4%). Haplotype diversity (*H*) varied from 0.370 (Costa Channel) to 0.872 (Falkland/Malvinas Islands). The number of haplotypes and polymorphic sites per locality ranged from 4 (Costa Channel) to 14 (Puerto Montt) and from 5 (Costa Channel) to 13 (Puerto Montt), respectively (Table 1). However, rarefaction analysis of the number of haplotypes using PAST [52] showed that most of the variations were the result of different sampling sizes, particularly in the case of Puerto Montt. The average number of nucleotide differences (*Π*) and the nucleotide diversity (π) were low in most of the localities with the exception of the Falkland/Malvinas Islands (Table 1).

**Table 1:** Number of individuals per locality, their respective diversity indices and neutrality tests results based on mtDNA (COI) sequences

**Locality**	***N***	***k***	***H***	***S***	***Π***	***π***	**Tajima’s *D***	**Fu’s *F***_***S***_
**Metri**	25	9	0.743	10	1.380	0.00206	−1.58	−4.27**
**Puerto Montt**	43	14	0.788	13	1.305	0.00194	−1.74*	−10.07***
**Concoto Island**	23	7	0.577	7	0.767	0.00114	−1.88*	−4.27*
**Puerto Aguirre**	24	6	0.496	6	0.790	0.00118	−1.53	−2.70*
**Costa Channel**	24	4	0.370	5	0.830	0.00124	−1.10	−0.24
**Serano Channel**	24	10	0.775	9	1.500	0.00224	−1.23	−5.34***
**London Island**	28	9	0.630	11	1.365	0.00203	−1.68	−3.99*
**Santa Ana**	24	10	0.775	11	1.652	0.00246	−1.48	−4.84**
**Posession Bay**	29	10	0.820	17	2.345	0.00349	−1.57	−2.54*
**Orange Bay**	24	11	0.819	14	1.906	0.00284	−1.72	−5.43**
**Tekenika Bay**	24	11	0.822	13	1.822	0.00272	−1.65	−5.70**
**Virginia Bay**	25	11	0.693	16	2.247	0.00335	−1.65	−4.34**
**Puerto Deseado**	27	6	0.650	8	1.738	0.00259	−0.50	−0.18
**Falkland Island**	13	7	0.872	14	5.205	0.00776	0.63	0.37
**COI Total**	357	57	0.761	59	1.936	0.00288	−2.30**	−68.58***

Where *n*: number of sampled specimens; *k*: number of haplotypes detected; *S*: polymorphic sites; *H*: haplotype diversity; Π: average number of nucleotide difference; π: nucleotide diversity *p < 0.05, **p < 0.01, ***p < 0.001. For graphical purposes Falkland/Malvinas Islands are in the table referred as Falkland Islands.

**Figure 1:**
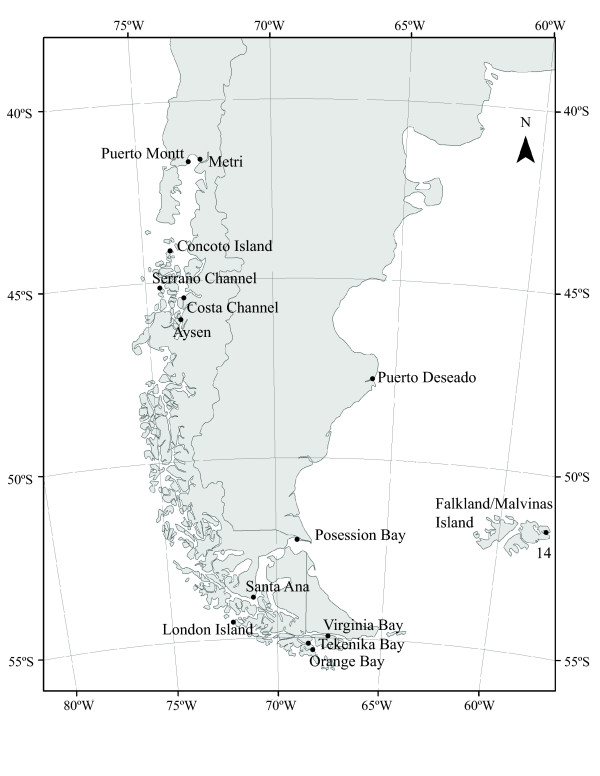
**Sampling localities of *N. magellanica *in Patagonia where:** 1) Puerto Montt (R.F.), 2) Metri (R.F.), 3) Concoto Island (Ch.A.), 4) Puerto Aguirre (Ch.A.), 5) Costa Channel (Ch.A.), 6) Serrano Channel (Ch.A.), 7) London Island (S.M.), 8) Santa Ana (S.M.) 9) Possession Bay (S.M.), 10) Tekenika Bay (C.H.), 11) Orange Bay (C.H.), 12) Virginia Bay (C.H.), 13) Puerto Deseado, 14) Falkland/Malvinas Islands. R.F.=Reloncaví Fjord; Ch.A.=Chonos Archipelago; S.M.=Strait of Magellan; C.H.=Cape Horn. * Significant values after Bonferroni correction.

The median-joining network depicted from the COI data set exhibited 56 different haplotypes (Figure 1). In the Pacific sector we observed a typical star-like topology in which the central haplotype (H1) was the most frequent (> 50%) and widely distributed. As proposed by Posada & Crandall [53] this haplotype should represent the most ancestral one, whereas the most derived ones are related to it with a maximum branch length of twelve mutational steps (H55 and H52). Two haplotypes H3 and H42, located no more than two mutational steps away from H1, were present in several localities and showed intermediate frequencies (H3=9.5% and H42=5.3%; Additional files 1 and 2). We did not detect an association between haplotype identity and geographical locality in Pacific Patagonia. Several haplotypes (H2, H8, H12, H15, H26, and H30) were present in more than five individuals belonging to different localities. The remaining haplotypes occurred at low frequencies and we identified 29 singletons. However, in Puerto Deseado H41 and H42 were the most common haplotypes, while H1 (the most frequent in Pacific Patagonia) was present in a single individual (Figure 1). We found some degree of haplotype similarity of haplotype frequencies among localities from the southern tip of the Pacific and Atlantic Patagonia. For instance, none of the dominant haplotypes in Puerto Deseado (H41 and H42) were found in the Reloncavi Fjord, Chonos Archipelago, and Pacific localities in the Strait of Magellan but these haplotypes were observed in Cape Horn sites and in Possession Bay at the eastern mouth of the Strait of Magellan. Finally, in the Falkland/Malvinas Islands we detected two groups of haplotypes. The first includes haplotypes closely related to (H54 and H56) and even shared with (H1 and H26) Patagonian diversity, while the second consists of private haplotypes, separated from the others by approximately 10 mutational steps (Figure 1).

SAMOVA analyses in the whole data set recovered two maximally differentiated groups explaining 51.73% of the total variation. These groups were 1) the Falkland/Malvinas Islands and 2) the rest of the localities along Pacific and Atlantic Patagonia. In a second SAMOVA analysis excluding the Falkland/Malvinas Island sample it recognized two maximally differentiated groups explaining 37.63% of the total variation named: a) Pacific Patagonia (including Reloncaví Fjord, Chonos Archipelago, the Strait of Magellan and Cape Horn localities), and b) Puerto Deseado (Atlantic Patagonia). Finally, in a third SAMOVA analysis including only Pacific and Cape Horn localities it did not recover significant spatial structure in the species. Evidence of this are the levels of variance among groups that explained just between 3.3% and 4.7% of the total variation, while within localities differences represented 95% to 97.5%. According to this, we recognized three main groups named: a) Pacific Patagonia including Reloncaví Fjord, Chonos Archipelago, Strait of Magellan and Cape Horn localities, b) Atlantic Patagonia including Puerto Deseado and c) the Falkland/Malvinas Islands. General differentiation coefficient measured over 14 populations of *N. magellanica* was low, especially taking into account average G_ST_=0.126 and N_ST_=0.190. Even when some pairwise comparisons between Pacific Patagonia localities showed significant levels of genetic structure (Table 2), none of them were statistically significant after Bonferroni correction. The permutation test indicated that N_ST_ is significantly higher than G_ST_ (P < 0.05), pointing to a phylogeographical structure for *N. magellanica* mtDNA haplotypes between the Falkland/Malvinas Islands and the rest of the localities.

**Table 2:** **G**_**ST **_**(below diagonal) and N**_**ST **_**(above diagonal) pairwise comparisons among the analyzed sites in Patagonia**

	**1**	**2**	**3**	**4**	**5**	**6**	**7**	**8**	**9**	**10**	**11**	**12**	**13**	**14**
1		0.0115	0.0252	0	0	0	0.0095	0	0.0041	0.0263	0.0025	0.0395	**0.3880**	**0.4387**
2	0.0014		0	0.0020	0.0110	0	0.0216	**0.0339**	**0.0346**	**0.0364**	**0.0281**	**0.0609**	**0.4049**	**0.4876**
3	0.0210	0.0105		0	0.0228	0.0156	0.0112	**0.0479**	**0.0577**	0.0284	0.0214	**0.0596**	**0.4298**	**0.4590**
4	0.0183	0.0370	0		0	0.0038	0	0.0066	0.0298	0.0197	0.0021	0.0399	**0.4274**	**0.4693**
5	**0.0638**	**0.0786**	0.0070	0		0.0015	0.0019	0	0.0139	0.0249	0.0054	0.0385	**0.4263**	**0.4636**
6	0	0	0.0122	0.0233	**0.0711**		0.0119	0.0067	0	0.0307	0.0135	0.0372	**0.3824**	**0.4211**
7	0.0297	**0.0268**	0	0.0088	0.0358	0.0254		0	**0.0389**	0	0	0.0120	**0.3865**	**0.4280**
8	0	0	0.0250	0.0284	**0.0711**	0	0.0172		0.0086	**0.0250**	0	0.0059	**0.3733**	**0.4131**
9	0.0018	0	0.0447	**0.0694**	**0.1168**	0	**0.0579**	0		0.3963	0.0235	0.0332	**0.2631**	**0.3967**
10	0.0092	0.0083	0.0387	**0.0600**	**0.1063**	0.0062	0.0197	0.0040	0.0097		0	0.0064	**0.3206**	**0.3661**
11	0	0.0025	0.0314	0.0480	**0.0965**	0	0.0112	0	0.0041	0		0	**0.3408**	**0.3831**
12	0.0067	0.0693	0	0.0027	0.0359	0.0032	0	0.0009	0.0248	0.0091	0.0072		**0.3332**	**0.3416**
13	**0.2862**	**0.2613**	**0.3691**	**0.4065**	**0.4680**	**0.2719**	**0.3457**	**0.2719**	**0.1993**	**0.2216**	**0.2350**	**0.2998**		**0.5134**
14	**0.1334**	**0.1143**	**0.2093**	**0.2548**	**0.3298**	**0.1175**	**0.1835**	**0.1175**	**0.1037**	**0.0891**	**0.0902**	**0.1487**	**0.2486**	

Where 1) Metri; 2) Puerto Montt; 3) Concoto Island; 4) Puerto Aguirre; 5) Costa Channel; 6) Serrano Channel; 7) London Island; 8) Santa Ana; 9) Posession Bay; 10) Orange Bay; 11) Tekenika Bay; 12) Virginia Bay; 13) Puerto Deseado; 14) Falkland/Malvinas Islands. Statistically significant differences after 50,000 iterations (p < 0.05) are marked in bold.

**Figure 2:**
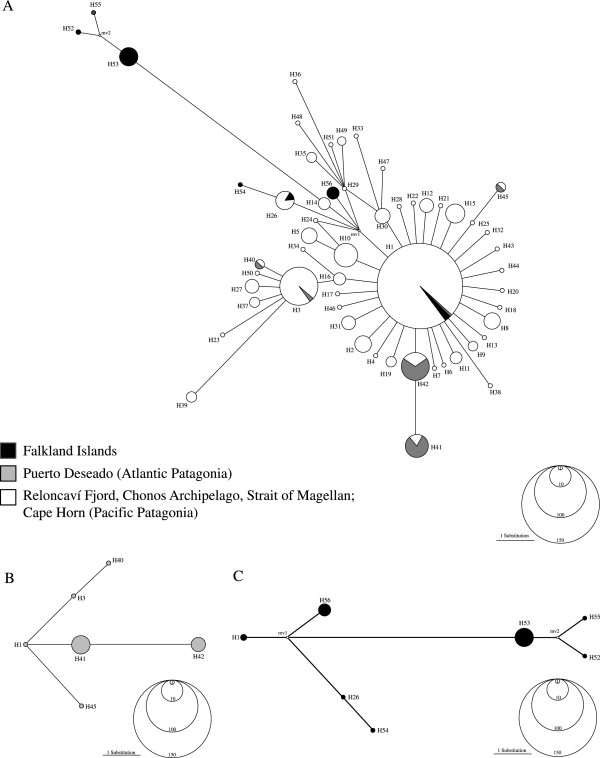
**Haplotype network including 357 *Nacella magellanica *mtDNA COI sequences. **Each haplotype is represented by a colored circle indicating where it was collected; the size of the circle is proportional to its frequency in the whole sample. mv=median vector (theoretical haplotype that has not been collected but should exist).

Migration rates among SAMOVA’s defined groups (Pacific Patagonia, Atlantic Patagonia and Falkland/Malvinas Islands) showed clear evidence of asymmetrical gene flow. The total number of immigrants per generation (N_e_m) from the Pacific to the Atlantic was 16.6, while from the Pacific to the Falkland/Malvinas Islands gene flow was lower, N_e_m=2.21 (Figure 2). In contrast, gene flow from the Atlantic to the Pacific was low (N_e_m=0.09) and from the Atlantic to the Falkland/Malvinas Islands even lower (N_e_m=0.0004). Similarly, the migration rate from the Falkland/Malvinas Islands to the Pacific was 0.26 and from the Falkland/Malvinas Islands to Atlantic Patagonia was extremely low, 0.0002 (Figure 2). We detected a small but significant correlation between genetic and geographic distances when all the analyzed localities were included (r=0.37; P < 0.001). This result is expected, considering that most of the significant pairwise comparisons (G_ST_=69% and N_ST_=71%) included Puerto Deseado (Atlantic Patagonia) and Falkland/Malvinas Islands (Table 2). Considering this, we performed a new Mantel test including only Pacific Patagonia localities and the analysis did not detect significant correlation (r=0.14; P=0.10) between geographic and genetic distance from the Reloncaví Fjord to Cape Horn.

Tajima’s *D* and Fu’s *F*_*S*_ neutrality tests showed contrasting results among the three defined groups in *N. magellanica* of Patagonia. These indices were negative and highly significant in Pacific Patagonia, while in Atlantic Patagonia they were negative but not significant. Finally, in the Falkland/Malvinas Islands both indices were positive and not significant, pointing to different demographic histories among the analyzed sectors. Similarly, the distribution of pairwise differences varied considerably among the recognized genetic groups in *N. magellanica*. For instance, the mismatch distribution in Pacific Patagonia was L-shaped (Figure 2), in Atlantic Patagonia it showed a bimodal pattern (Figure 2) and in the Falkland/Malvinas Islands it had a multimodal distribution (Figure 2).

**Figure 3:**
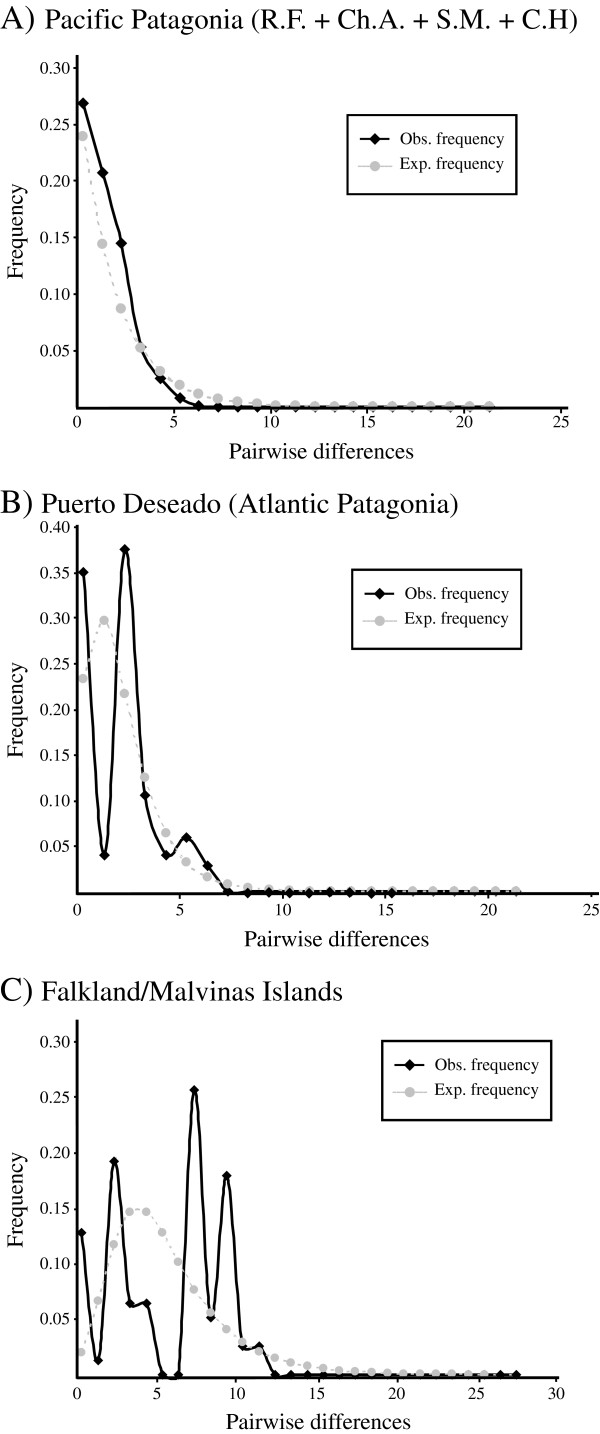
**Pairwise difference distribution (mismatch distribution) for the Cytochrome c oxidase subunit I (COI) in *N. magellanica *in different areas of Patagonia.A)** Pacific Patagonia; **B)** Puerto Deseado; **C)** Falkland/Malvinas Islands. R.F.=Reloncaví Fjord; Ch.A.=Chonos Archipelago; S.M. Strait of Magellan; C.H. Cape Horn.

Recent molecular studies recognized an error in the use of substitution rates inferred from phylogenetic analyses in studies at the population level [54-57]. It has been demonstrated in different groups of organisms that short-term mutation rates may be tenfold higher than long-term rates [56-58]. Including this tenfold correction to the specific molecular rate estimated for nacellids [51] and used in another study in the species [49], the start of the expansion in *N. magellanica* under a sudden growth model occurred ~ 6.3 ka. The Bayesian skyline plot analysis indicates that the most common recent ancestor of the current *N. magellanica*’s diversity occurred ~ 24.1 ka while population expansion began around 9 ka (Figure 3).

## Discussion

Understanding how ecosystems and species respond to climate change has become a major focus of ecology and conservation biology [59-62]. One of the central premises of biogeography is that climate exerts a dominant control over the distribution of species [63,64]. Evidence from the fossil record [65,66] and from reconstructions based on molecular data [59,67,68] have demonstrated that changing climate generates a profound influence on species’ range expansion and contraction, as well as in their patterns of genetic diversity and structure [3,6,7,10,11, 68-71]. In the particular case of *Nacella magellanica* it may be possible to ascribe the observed patterns of genetic diversity and structure to drastic demographic effects of the glacial cycles on the species in its distribution in Patagonia. For instance, COI diversity in the species are lower than those observed in temperate patellogastropods [72-74] but higher than in its Antarctic relative, *N. concinna*[51]. These results agree with molecular studies in Northern Hemisphere biota where the impact of the Quaternary glacial cycles exerted stronger effects in the demography of marine benthic populations at higher latitudes and particularly in polar regions [75].

### Genetic homogeneity and recent population expansion in *N. magellanica* in Pacific Patagonia

According to Camus [76], the Chilean coast of Pacific Patagonia can be considered as a major insular system that includes many islands, gulfs, peninsulas, fjords, and channels that generate a very complex landscape as a result of the marked climatic changes during the Quaternary. Despite such a complex landscape, we found a single genetic unit in *N. magellanica* from the Reloncaví Fjord (41.5°S) to Cape Horn (55.9°S), an area that includes ~ 1300 km in a straight line but about 84,000 km of irregular coasts [77-80]. For marine benthic organisms, duration of planktonic larval stages is expected to correlate with dispersal ability [81,82]. Regretfully, there is no direct information about larval duration in the species, but it is expected that its development should be similar to the Antarctic limpet, with a free-swimming planktonic period for 1 to 2 months in the water column [83,84]. Consequently, it could be expected that the *N. magellanica* larval period extends for at least four weeks, considering the effect of temperature on development and metabolism [85,86]. Gene flow mediated by larval dispersal may have been enhanced by oceanographic conditions in the area and constitutes a suitable explanation for the low levels of genetic diversity and the high degree homogeneity in the *N. magellanica* populations from Pacific Patagonia. Even more, considering the observations of *N. magellanica* in holdfasts of *M. pyrifera*[48], rafting could also constitute an important dispersal mechanism, particularly for the colonization of geographically distant areas.

The general genetic pattern of *N. magellanica* along its distribution in Pacific Patagonia indicates low levels of nucleotide diversity and number of nucleotide differences. On one hand, the lowest values were found at localities that should have been severely ice-impacted during the LGM including channels and fjords such as Costa Channel, Puerto Aguirre, and Concoto Island. On the other hand, higher diversity levels were found at northern localities (Metri and Puerto Montt), more oceanic areas (Serrano Channel and London Island), the Strait of Magellan and Cape Horn. In spite of these slight diversity differences among localities, theory predicts that large population sizes should maintain high levels of genetic variability, because genetic drift is low and the mutational rate is high. General molecular diversity indices estimated in *N. magellanica* in the Pacific (θ_*k*_=4.84; θ_*S*_=2.93; θ_*H*_=2.41; θ*π*=1.77) would be sustained by effective sizes (N_e_) between 133,750 and 372,000 individuals. These estimations are smaller by far than the expected population sizes in the species, considering the high densities reported [41,42,44,87-89]. Low levels of genetic diversity together with dominant haplotypes widely distributed are consistent with the hypothesis of a recent range expansion [90,91]and high levels of migration [3,70]. Moreover, significant negative Tajima’s *D* and Fu’s *F*_*S*_ indices, together with a unimodal mismatch distribution in Pacific Patagonia are the result of an excess of low frequency haplotypes, commonly explained by recent demographic processes.

Traditional genetic models of glacial refugia and routes of recolonization include the prediction of low genetic diversity in formerly glaciated areas with a small number of haplotypes dominating disproportionally large areas, and high diversity in glacial refugia [2,7,71]. Based on the patterns of genetic structure in *N. magellanica*, the hypothesis of persistence of the species in multiple glacial refugia along Pacific Patagonia followed by expansion from surviving populations is most unlikely. If these periglacial populations experienced strong bottlenecks during the LGM, they may exhibit low genetic diversity as expected in recolonized areas with no refugia, but should have more endemic diversity than recently recolonized areas [71]. Even in the presence of high levels of gene flow under a multiple *in situ* refugia hypothesis, it is expected to find more than one haplotype exhibiting high frequency and each of these haplotypes could derive from a putative glacial refuge. However, in the case of *N. magellanica*, coupled with the low levels of nucleotide diversity we found an absence of genetic differentiation along Pacific Patagonia with just one dominant haplotype (H1). The lack of structure in a large geographical area with a single dominant haplotype, the absence of correlation between geographic and genetic distance and the evidence of recent demographic growth support the hypothesis of a recent expansion in the species, possibly mediated by its indirect development. Such larval-mediated postglacial recolonization processes in the Northern Hemisphere have been frequently recognized in marine organisms [70,92,93]. In contrast, few studies have established the importance of the developmental mode in the phylogeographic patterns of marine invertebrates in Patagonia. Absence of genetic structure, as observed in *N. magellanica* in Pacific Patagonia, have been also recognized in other marine organisms with indirect development, including the mytilid *Mytilus edulis*[94], fishes like *Eleginops maclovinus*[19] and *Sebastes oculatus*[95], and in macroalgae including *Durvillaea antartica*[15] and *Macrocystis pyrifera*[38]. These studies contrast with the results obtained in the direct developer *Acanthina monodon* that exhibits marked differentiation between northern and southern Pacific Patagonia localities [39]. The patterns of genetic structure observed in different groups of marine organisms across Patagonia further support the importance of the developmental mode and the prevailing directions of currents and winds.

According to Hein *et al.*[96] the timing of the LGM extent and the onset of deglaciation occurred broadly synchronously throughout Patagonia. In northern areas of Pacific Patagonia the final ice advance is dated about 17.9 ka [97,98] and warming began at 17.5 ka [99]. Similarly, around the Strait of Magellan the final ice advance occurred prior to ca. 17 ka [100] while a major and rapid warming period occurred between 14–10 ka [23,100, 101]. Pollen statrigraphic studies in the Beagle Channel and Tierra del Fuego suggest that the disappearance of ice in that sector occurred ~ 11.6 ka [96-102]. Based on our estimations, population expansion in *N. magellanica* would have occurred ~ 6.3 ka under a sudden growth model and ~ 9.0 ka under the Bayesian Skyline Plot approximation. Estimated dates of population expansion in *N. magellanica* are consistent with previous analysis in the species [49] and with thermal records of warmer conditions in Patagonia. Also, paleontological studies on postglacial mollusk faunas in the northern coast of the Beagle Channel suggest that major expansion of taxa occurred after the glaciers receded fully (~ 10 ka). Under the relatively warmer conditions of the middle Holocene (5.0 to 4.0 ka), the fossil record indicates a process of diversification of several mollusk taxa including *Nacella*[103-105].

### Genetic differentiation among Pacific Patagonia, Atlantic Patagonia and the Falkland/Malvinas Islands

**Figure 4:**
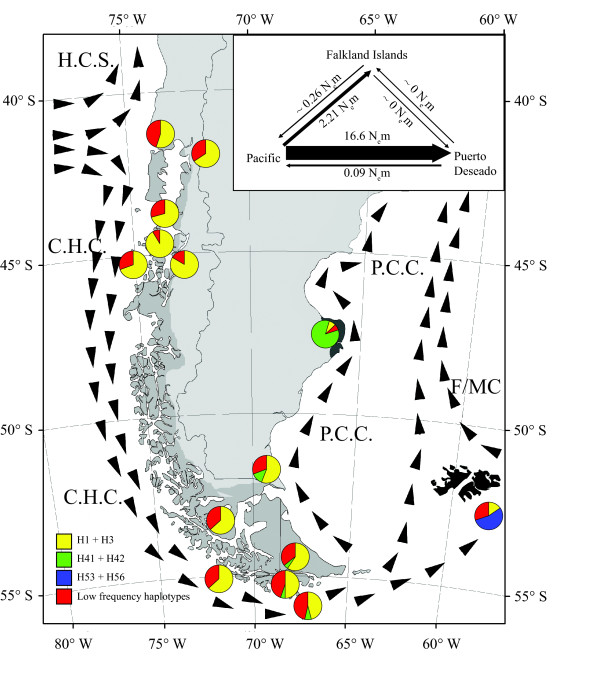
**Prevailing direction of currents and winds in southern South America and frequency of the dominant haplotypes in each locality.** H.C.S.=Humboldt Current System; C.H.C.=Cape Horn Current, M/FC=Falkland/Malvinas Current; P.C.C. Patagonian Coastal Current. Migration rate measured as effective number of migrants (N_e_m) among the main areas in Patagonia (Pacific Patagonia, Puerto Deseado and the Falkland/Malvinas Islands).

In spite that *N. magellanica* constitute a single panmictic unit along Pacific Patagonia, we detected clear differences among three main areas in Patagonia. Marked levels of genetic structure in the species among these areas may be explained by differences in their respective glaciological histories, rocky shore availability and the prevailing currents and winds among them. First, ice-shelf advances and retreats differentially affected Pacific and Atlantic Patagonia during Pleistocene glacial cycles. Pacific Patagonia was almost completely covered by ice during the LGM and shallow marine habitats should have been severely affected. In contrast Atlantic Patagonia was only affected over the piedmont areas to the east and to the current submarine platform south of Río Gallegos [96,106,107]. Sea levels changes during the LGM might have differentially affected Atlantic and Pacific populations. Atlantic and Falkland Island populations might have had even less rocky substrated than today, with exposure of the shelf, and would also have moved north and eastward during the LGM following the shorelines. Geomorphologic evidence in West Falkland/Malvinas suggests that during the Cenozoic the largest glaciers were no more than 2.7 km long [108]. According to this scenario, the Pacific population would have been more severely hampered during LGM, as suggested by significant negative Tajima and Fu’s tests and an L-shaped mismatch distribution. In contrast, populations from Atlantic Patagonia and Falkland/Malvinas Islands did not show deviation from the mutation-drift equilibrium model and also exhibited multimodal mismatch distributions, in agreement with the expectation for more stable populations (Figure 4). The results observed in Puerto Deseado did not match with those recently published by de Aranzamendi *et al.*[49] that detected signal of recent demographic expansion along Atlantic Patagonia. In this respect, contrasted demographic signals detected here between Pacific and Atlantic populations must be taken cautiously considering the differences in sample size [109]. However, the main signal detected here in the whole Pacific data set is also observed in each locality from the Reloncaví Fjord to Cape Horn and sample size in each one of them is comparable to the ones of Puerto Deseado and the Falkland/Malvinas Islands (Table 1). Second, the Atlantic coast includes less rocky shore ecosystems than Pacific Patagonia where more suitable rocky habitat are available for the species. These differences between Pacific and Atlantic Patagonia in terms of habitat availability, has been also recognized in comparative biodiversity studies in the southern tip of South America [78]. Off southern Chile the Cape Horn Current (CHC) flows southward around Cape Horn while the Malvinas-Falkland Current (M-FC) is a northward running branch of the former that moves about up to 28°S following the continental shelf margin [110-112]. The minor Patagonian Coastal Current (PCC) influences the Atlantic Patagonia coast and moves northward up to 38°S [110-112]. Moreover, oceanographic fronts such as the Atlantic Patagonian cold estuarine front on the eastern margin of the Strait of Magellan has been described as an oceanographic barrier between Pacific Patagonia and the southern Atlantic coast [113-115]. As expected under the general circulation pattern in this region, we found asymmetrical gene flow in the species among Pacific Patagonia, Atlantic Patagonia and the Falkland/Malvinas Islands. Migration rates to Pacific Patagonia from the Atlantic population and from the Falkland/Malvinas Islands were very low. Similarly, migration rates between these islands and Atlantic Patagonia were almost negligible. In this respect, it is proble that Atlantic Patagonia is continuously receiving haplotypes from the Pacific. In fact, haplotypes from Puerto Deseado are shared with Pacific Patagonia localities and especially with those from Tierra del Fuego, the Beagle Channel, and the eastern mouth of the Strait of Magellan Additional file 2. Considering the migration estimation from the Atlantic to the Pacific and the prevailing direction of the oceanic fronts and winds, the hypothesis of postglacial recolonization from Atlantic Patagonia to the Pacific is most unlikely. Similar patterns of genetic discontinuities between Pacific and Atlantic localities in Patagonia have been found in *Enteroctopus megalocyathus*[116] and in the scallop *Aequipecten tehuelchus*[117]. Moreover, de Aranzamendi *et al.*[49] found significant genetic differentiation between *N. magellanica* populations from Tierra del Fuego and northern localities such as Golfo San José and Golfo Nuevo.

In the Falkland/Malvinas Islands, even when we included only 13 individuals we detected the highest levels of genetic diversity. Furthermore, this population was characterized by positive Tajima’ *D* and Fu’s *F*_*S*_ indices, a multimodal mismatch distribution and an expanded genealogy (Figure 4). According to the Quaternary genetic model [2,3,7,71], the Falkland/Malvinas Islands could be considered as a glacial refugium, considering the higher level of genetic diversity and the presence of endemic haplotypes (H53, H52 and H55) clearly differentiated from the Patagonian diversity. Moreover, these islands have been previously proposed as refugial areas for several plant species during the LGM [21,108,118] and as relevant area for conservation [119].

However, even if our data support the persistence of *N. magellanica* in Falkland/Malvinas Islands during the LGM, they do not support a scenario of posterior recolonization from Falkland/Malvinas Islands to Atlantic and/or Pacific Patagonia. Considering our migration rate estimations, most of the gene flow in *N. magellanica* is derived from Pacific Patagonia to the other areas. The particular case of the Falkland/Malvinas Islands seems to represent a sink area where private surviving haplotypes are mixed together with recently arrived ones from Pacific Patagonia.

## Conclusions

Historical factors and life-history traits such as its indirect development play a main role in the connectivity of *N. magellanica*. In concert with the high dispersal potential of the species, we detected a rapid postglacial recolonization process in a very complex landscape likely related the deglaciation process along Pacific Patagonia. In contrast to the model of Pleistocene biogeography, where higher levels of genetic diversity are expected at lower latitudes, in *N. magellanica* we did not detect a clear relationship between latitude and genetic diversity. The absence of evidence of a progressive southward recolonization through recurrent founder effects may be the result of the synchronous deglaciation process along Pacific Patagonia [96] together with high dispersal capacities. In contrast, lower genetic diversity detected in the inland sea, characterized by fjords and channels, could indicate that these areas represent those most recently recolonized by *N. magellanica*. In this region, the timing of recolonization would therefore have followed a west to east trend, contrasting to the usual north–south model of Pleistocene biogeography in South America. At the same time, this study gives further evidence for the role of the major current systems among different areas of Patagonia through the existence of an asymmetrical pattern of gene flow from Pacific Patagonia to Atlantic Patagonia and the Falkland/Malvinas Islands following the CHC, the M-FC and the PCC. According to our results, *N. magellanica* persisted in the Falkland/Malvinas Islands during the Quaternary glacial cycles and therefore represents a relict population. However, the pattern of genetic diversity strongly suggests that this population did not participate to the postglacial recolonization of southern South America. Considering oceanic and atmospheric circulation in the province and the pattern of gene flow we found among Patagonian sectors, the Falkland/Malvinas Islands seem to represent a sink area where recently arrived and endemic haplotypes coexist. The main pattern of genetic diversity and structure in *N. magellanica* appears to be the result of the combination of the impact of the last glacial period in Pacific Patagonia and the prevailing oceanographic circulation, together with life-history traits like its indirect development and its narrow bathymetric range. These historical and contemporary processes may constitute important factors in shaping the modern biogeography of most shallow marine benthic invertebrates inhabiting the Patagonian Province.

Future research in *N. magellanica* will include a broader sampling effort along the Atlantic coast and the use of recently developed fast-evolving markers [120] in order to corroborate the observed pattern of genetic structure. Finally, more studies on other species of *Nacella* as well as other marine benthic taxa are required in order to provide a better understanding of the historical and recent processes governing the patterns of genetic structure and connectivity in southern South America. This information will provide an empirical framework in order to generalize the postglacial biogeographic model proposed here for the limpet *N. magellanica*.

## Methods

### Sample collection, DNA extraction, PCR amplification and sequencing

**Figure 5:**
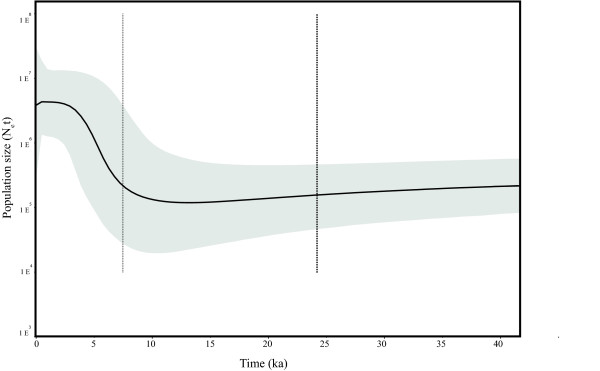
**Historical demographic trends of the effective population size (Ne) constructed using a Bayesian skyline plot approach based on Cytochrome oxidase subunit I (COI) haplotypes of *N. magellanica. ***The *y*-axis is the product of effective population size (N_e_) and generation length in a log scale while the *x*-axis is the time in 10^3^ before present. The median estimate (black solid line) and 95% highest probability density (HPD) limits (grey) are shown. The thick dashed line represents the time of the most recent ancestor (*trcma*) and the thin dashed line represents time for the expansion in the species.

Individuals were collected between 2007–2011 from the intertidal zone in 14 localities (Figure 5). Along Pacific Patagonia we included two localities in the northern limit of the species distribution in the Reloncaví Fjord (41.5°S), four localities from the Chonos Archipelago (44° - 46°S), two localities in the Strait of Magellan (52° - 53°S) and three localities from the Cape Horn Biosphere Reserve (54° - 55°S; Figure 5). We also included in the analyses two localities in Atlantic Patagonia, Puerto Deseado (47°45’ S; 65°52’ W) and Possession Bay (52°17’ S; 68°57’ W) in the eastern mouth of the Strait of Magellan. Finally, we included in the analyses 13 individuals from the Falkland/Malvinas Islands (51°41’ S; 57°50 W; Figure 5). Specimens were identified based on shell morphology, sculpture, height, and coloration [40] and with the help of diagnostic external characteristic of the species such as coloration of the foot muscle and the mantle tentacle [43]. Individuals were fixed in ethanol (95%) and whole DNA was extracted from the mantle using the salting-out method described by [121]. PCR amplifications of a partial fragment of the mtDNA gene Cytochrome c Oxidase Subunit I (COI) were performed using the universal primers described by Folmer *et al.*[122] and PCR conditions were done following [45]. Double stranded PCR products were purified using the QIAquick Gel Extraction Kit (QUIAGEN), and sequenced in both directions using an Automatic Sequencer ABI3730 x 1 at Macrogen, Inc. (Seoul, Korea).

### Analyses

DNA chromatograms were manually edited using Proseq v. 2.91 [123] and aligned with ClustalW [124]. COI sequences were translated to amino acids to check for sequencing errors and/or the presence of pseudogenes with MEGA 5.0 [125]. We performed a DNA saturation analysis following Roe & Sperling [126] to evaluate the levels of saturation changes along the *N. magellanica* COI data set. New COI sequences have been submitted to GenBank database (Accession Numbers: JX262742 – JX262797).

We estimated the levels of polymorphism in *N. magellanica* using standard diversity indices, haplotype number (*k*), the number of segregating sites (*S*) and haplotypic diversity (*H*) for each locality and for the whole COI data set using DnaSP v.5.00.07 [127]. We also estimated average pairwise sequence differences (*Π*) and nucleotide diversity (*π*). Population parameters (Tajima’s *D* and Fu’s *F*_*S*_) were calculated for all populations using DnaSP and Arlequin v.3.11 [128].

Genetic differentiation was determined in two ways following [129,130] using mean pair-wise differences (N_ST_) and through their haplotype frequencies (G_ST_) in Arlequin. We performed permutation tests (25,000 random iterations) of both coefficients to confirm statistical differences among the analyzed localities. Moreover, both parameters were compared with Permut (http://www.pierroton.inra.fr/genetics/labo/Software/) using an analytical test. We tested whether N_ST_ >> G_ST_ by comparison of the N_ST_ values measured directly with those obtained after 1000 random permutation of haplotype identities [131]. Using SAMOVA v.1 (Spatial Analysis of Molecular Variance) [132] we defined the number and composition of geographically homogeneous, maximally differentiated groups of localities. This method aims to maximize the proportion of total genetic variance due to differences among groups minimizing the variance portion among population within groups. Once these groups were defined, we estimated the levels of migration among them using a Markov Monte Carlo coalescent genealogy sampler implemented in LAMARC v.2.1.8 (Likelihood Analysis with Metropolis Algorithm using Random Coalescence) [133]. This approximation allows to estimate migration levels among the recognized groups of *N. magellanica* and at the same time to test whether the migration was symmetric or asymmetric among them. We examined the significance of the correlation between genetic divergence measured as Slatkin’s linearized F_ST_ [Phi_ST_/(1 - Phi_ST_)] and geographical distance between localities using a Mantel test implemented in Arlequin; associated probabilities were estimated with 25,000 permutations.

We reconstructed genealogical relationships for *N. magellanica* using median-joining haplotype networks in Network v.4.6 (http://www.fluxus-engineering.com) [134]. To estimate past population dynamics in the species within Pacific Patagonia we applied two methods. First, we used the sudden population growth model [91] which rests on the assumption that population growth and decline events leave characteristic signatures in the distribution of nucleotide site differences between pairs of individuals. We constructed the distribution of pairwise differences (mismatch distribution) in *N. magellanica* to determine whether *N. magellanica* has undergone sudden population growth. We compared the distribution of pairwise differences in *N. magellanica* with expectations of a sudden expansion model. Three main parameters were estimated: i) the date of growth/decline (*τ=2μt*) measured in units of 1/2 *μ* generations where t=time in years and *μ=*mutation rate per sequence per generation, initial population size (*Θ*_*i*_) before the population growth/decline and a final theta (*Θ*_*f*_) after population growth/decline. These demographic expansion parameters were determined using a nonlinear least squares approach implemented in Arlequin [135]. The goodness of fit between the observed and expected mismatch distributions was tested using a parametric bootstrap approach that uses the sum of squared deviations as a statistic test implemented in Arlequin. Second, we used a Bayesian skyline plot method implemented in BEAST v. 1.6 [136], which detects demographic signatures from nucleotide sequences that are not readily described by simple demographic models [137,138]. We analyzed the data set under an uncorrelated lognormal relaxed molecular clock model using an evolutionary rate of 1.1% per million years estimated for COI in nacellids [51], using the GTR + G + I model previously estimated with MrModeltest v.2.3 (http://www.abc.se/~nylander/) and a piecewise constant Bayesian skyline model with 10 groups. Before choosing this model we performed Bayesian Skyline Plot analyses using *N. magellanica*’s COI data set with three different models: an uncorrelated lognormal relaxed clock, an uncorrelated exponential relaxed clock, and a strict molecular clock. Estimated bayes factors among these models strongly supported the molecular clock hypothesis. We ran the analyses for 350 × 10^6^ generations, making sure that the effective sampling sizes for each statistic were at least 1500. Convergence was examined in Tracer v. 1.5 (http://beast.bio.ed.ac.uk/Tracer) [137].

## Competing interests

The authors declare that they have no competing interests.

## Authors’ contributions

CG-W and E.P generated the ideas and design of the study, and all the authors took part in sample collections. CG-W produced the molecular data, and CG-W and E.P were responsible for data analyses. CG-W drafted the original version of the manuscript, and CG-W and E.P produced subsequent versions. All authors participated in a critical review of the manuscript and approved the final version for submission.

## Supplementary Material

Additional file 1 **A) Haplotype network including 357 *Nacella magellanica *mtDNA COI sequences.** Each haplotype is represented by a circle and its size is proportional to its frequency in the whole data set. mv=median vector (theoretical haplotype that has not been collected but should exist). B) Haplotype Network Puerto Deseado; C) Haplotype Network Falkland/Malvinas Islands. Click here for file

Additional file 2 Number of individulas presenting each haplotype and their corresponding locality. Click here for file
