# Supplementary material for: Towards a model of postglacial biogeography in shallow marine species along the Patagonian Province: lessons from the limpet Nacella magellanica (Gmelin, 1791)
Source: BMC Evol Biol. 2012 Aug 7;12:139. doi: 10.1186/1471-2148-12-139 (PMC3582430; doi:10.1186/1471-2148-12-139)
Supplement: Additional file 2 — Number of individulas presenting each haplotype and their corresponding locality. [file 1471-2148-12-139-S2.docx]

| Haplotype/Locality | 1 | 2 | 3 | 4 | 5 | 6 | 7 | 8 | 9 | 10 | 11 | 12 | 13 | 14 |
| --- | --- | --- | --- | --- | --- | --- | --- | --- | --- | --- | --- | --- | --- | --- |
| H1 | 12 | 19 | 15 | 17 | 19 | 12 | 17 | 11 | 11 | 10 | 10 | 14 | 1 | 2 |
| H2 | 2 | - | 1 | 1 | - | 1 | - | - | - | - | 1 | - | - | - |
| H3 | 5 | 4 | 1 | 3 | 3 | 4 | - | 4 | 5 | 1 | 2 | 1 | 3 | - |
| H4 | 1 | - | - | - | - | - | - | - | - | - | - | - | - | - |
| H5 | 1 | 2 | - | - | - | 2 | - | - | - | - | - | - | - | - |
| H6 | 1 | - | - | - | - | - | - | - | - | - | - | - | - | - |
| H7 | 1 | - | - | - | - | - | - | - | - | - | - | - | - | - |
| H8 | 1 | - | - | - | - | - | 1 | - | - | 2 | 2 | - | - | - |
| H9 | 1 | - | - | - | - | - | - | 1 | - | - | - | - | - | - |
| H10 | - | 5 | 2 | - | - | 1 | - | - | 4 | - | - | - | - | - |
| H11 | - | 2 | - | - | - | - | - | - | - | 1 | - | - | - | - |
| H12 | - | 2 | - | 1 | - | - | - | - | - | - | - | 1 | - | - |
| H13 | - | 1 | - | - | - | - | - | - | - | - | - | - | - | - |
| H14 | - | 1 | 1 | - | 1 | - | - | - | - | - | - | - | - | - |
| H15 | - | 2 | 2 | 1 | - | 1 | 1 | - | - | - | - | - | - | - |
| H16 | - | 1 | - | - | - | 1 | - | 1 | - | - | - | - | - | - |
| H17 | - | 1 | - | - | - | - | - | - | - | - | - | - | - | - |
| H18 | - | 1 | - | - | - | - | - | - | - | - | - | - | - | - |
| H19 | - | 1 | - | - | - | - | - | - | - | - | 1 | - | - | - |
| H20 | - | 1 | - | - | - | - | - | - | - | - | - | - | - | - |
| H21 | - | - | 1 | - | - | - | - | - | - | - | - | - | - | - |
| H22 | - | - | - | 1 | - | - | - | - | - | - | - | - | - | - |
| H23 | - | - | - | - | 1 | - | - | - | - | - | - | - | - | - |
| H24 | - | - | - | - | - | 1 | - | - | - | - | - | - | - | - |
| H25 | - | - | - | - | - | 1 | - | - | - | - | - | - | - | - |
| H26 | - | - | - | - | - | - | 1 | - | - | 3 | 2 | 1 | - | 1 |
| H27 | - | - | - | - | - | - | 3 | - | - | - | 1 | - | - | - |
| H28 | - | - | - | - | - | - | 1 | - | - | - | - | - | - | - |
| H29 | - | - | - | - | - | - | 1 | - | - | - | - | - | - | - |
| H30 | - | - | - | - | - | - | 1 | 1 | - | 1 | 1 | - | - | - |
| H31 | - | - | - | - | - | - | 2 | 2 | - | - | - | - | - | - |
| H32 | - | - | - | - | - | - | - | 1 | - | - | - | - | - | - |
| H33 | - | - | - | - | - | - | - | 1 | - | - | - | - | - | - |
| H34 | - | - | - | - | - | - | - | 1 | - | - | - | - | - | - |
| H35 | - | - | - | - | - | - | - | 1 | - | - | - | 1 | - | - |
| H36 | - | - | - | - | - | - | - | - | 1 | - | - | - | - | - |
| H37 | - | - | - | - | - | - | - | - | 1 | 1 | - | - | - | - |
| H38 | - | - | - | - | - | - | - | - | 1 | - | - | - | - | - |
| H39 | - | - | - | - | - | - | - | - | 1 | - | - | 1 | - | - |
| H40 | - | - | - | - | - | - | - | - | 1 | - | - | - | 1 | - |
| H41 | - | - | - | - | - | - | - | - | 2 | - | - | - | 10 | - |
| H42 | - | - | - | - | - | - | - | - | 2 | 1 | 1 | 1 | 13 | - |
| H43 | - | - | - | - | - | - | - | - | - | 1 | - | - | - | - |
| H44 | - | - | - | - | - | - | - | - | - | 1 | - | - | - | - |
| H45 | - | - | - | - | - | - | - | - | - | 1 | - | - | 1 | - |
| H46 | - | - | - | - | - | - | - | - | - | - | 1 | - | - | - |
| H47 | - | - | - | - | - | - | - | - | - | - | 1 | - | - | - |
| H48 | - | - | - | - | - | - | - | - | - | - | - | 1 | - | - |
| H49 | - | - | - | - | - | - | - | - | - | - | - | 2 | - | - |
| H50 | - | - | - | - | - | - | - | - | - | - | - | 1 | - | - |
| H51 | - | - | - | - | - | - | - | - | - | - | - | 1 | - | - |
| H52 | - | - | - | - | - | - | - | - | - | - | - | - | - | 4 |
| H53 | - | - | - | - | - | - | - | - | - | - | - | - | - | 1 |
| H54 | - | - | - | - | - | - | - | - | - | - | - | - | - | 1 |
| H55 | - | - | - | - | - | - | - | - | - | - | - | - | - | 1 |
| H56 | - | - | - | - | - | - | - | - | - | - | - | - | - | 3 |
| Total | 25 | 43 | 23 | 24 | 24 | 24 | 28 | 24 | 29 | 24 | 24 | 25 | 27 | 13 |

Where: 1) Metri; 2) Puerto Montt; 3) Concoto Island; 4) Aysen; 5) Costa Channel; 6) Serrano Channel; 7) London Island; 8) Santa Ana; 9) Posession Bay; 10) Orange Bay; 11) Tekenika Bay; 12) Virginia Bay; 13) Puerto Deseado; 14) Falkland/Malvinas Islands.
